# Figure-Disembedding Is Inferior in Non-autistic Compared to Autistic Individuals but Can Be Improved by Training

**DOI:** 10.3389/fpsyg.2022.857630

**Published:** 2022-07-25

**Authors:** Christine M. Falter-Wagner, Carola Bloch, Marta Robles, Lea Horch, Kai Vogeley, Alexandra Livia Georgescu

**Affiliations:** ^1^Department of Psychiatry and Psychotherapy, Medical Faculty, LMU Munich, Munich, Germany; ^2^Department of Psychiatry and Psychotherapy, University Hospital of Cologne, Cologne, Germany; ^3^Department of Clinical and Health Psychology, Autonomous University of Barcelona, Barcelona, Spain; ^4^Institute of Neuroscience and Medicine, Cognitive Neuroscience (INM-3), Research Centre Jülich, Jülich, Germany; ^5^Department of Psychology, Institute of Psychiatry, Psychology and Neuroscience, King’s College London, London, United Kingdom

**Keywords:** Autism Spectrum Disorder, local processing, priming, figure-disembedding, Gestalt

## Abstract

**Background:**

Figure-disembedding is one of the most discussed visuo-cognitive functions, in which individuals with Autism Spectrum Disorder (ASD) have been reported to outperform non-autistic individuals. A local processing bias has been assumed to underlie such superior performance patterns. The aim of the current study is to investigate whether processing preferences can be modified by procedural priming.

**Method:**

The current study used a procedural priming task (Navon figures) to induce more local or global processing in 25 autistic and 21 typically developing (TD) control participants, using hierarchical figures preceding the figure-disembedding task.

**Results:**

Participants with ASD outperformed non-autistic individuals in the unprimed baseline task version. The performance was not modulated by priming in either direction (toward a local or global processing style) in both groups. However, the performance of TD control participants was improved by training to the same level as that observed in the ASD group.

**Conclusion:**

Figure-disembedding performance in ASD is superior to that in TD control participants and robust against procedural priming or training. In contrast, performance in the TD control group can be improved up to the level of the ASD group. Any studies reporting superiority in individuals with ASD in figure-disembedding should consider training effects when evaluating group differences.

## Introduction

In the past decades, autism researchers have begun to investigate strengths in the cognitive profile of individuals with Autism Spectrum Disorder (ASD). This may have been inspired by the seminal work by Shah and Frith (1983, 1993), who showed superior performance in autistic compared to typically developing (TD) children on the block design sub-components of the Wechsler intelligence scales (Wechsler, 1981) and the Children’s Embedded-Figures Task (Witkin et al., 1971). The authors noted that a key characteristic of both tasks was that the detail-focused and “local” performance benefit in ASD might have been based on a disruption of Gestalt grouping. As a result, the salience of parts (local processing) over wholes (global processing) might increase as distraction from the overall Gestalt is reduced. This could have caused an advantage for the autistic over the TD group (e.g., Falter et al., 2008), which is in accordance with the “Weak-Central-Coherence” theory (WCC, Frith, 1989) predicting superior local processing (Happé and Frith, 2006) and originally also reduced global processing (Frith, 1989). The assumption that autistic persons have a preference for local processing has indeed been widely supported (Mottron et al., 1999; Behrmann et al., 2006; Wang et al., 2007). Supporting this view, the results of a meta-analysis that included 35 studies comparing among others performance on Block Design and Figure-Disembedding found that autistic participants are, on average, superior at tasks that require detail-focused processing compared to control persons (Muth et al., 2014). On the other hand, the second (original) assumption of the WCC theory, concerning the corresponding global processing impairments, has been challenged (Brosnan et al., 2004; Wang et al., 2007; Scherf et al., 2008; Koldewyn et al., 2013; Hadad and Ziv, 2015; Booth and Happé, 2018). Indeed, some studies report that autistic individuals can successfully re-organize the integral structure of visual information. For example, researchers have found superior performance of autistic individuals on tasks such as conjunctive visual search, where a target needs to be identified among distractors (Plaisted et al., 1998). Moreover, Dakin and Frith’s (2005) review article provides evidence only for superior local processing in persons with ASD, but not for impaired global processing. In response to this, alternative accounts of the WCC have been proposed, such as the Enhanced Perceptual Functioning account (EPF, Mottron and Burack, 2001; Mottron et al., 2006) and the Reduced Generalization Account (RG, Plaisted, 2001). According to EPF, the local preference of autistic individuals is not a consequence of a global deficit but merely of superior perceptual processes. It further suggests that local preference can be overcome by global requirements, and that the ability to process globally is not restricted. Moreover, the “low-level” perceptual processes are characterized by an atypical autonomy with reduced influence by “high-level” processes (Mottron and Burack, 2001; Mottron et al., 2006). The RG account assumes that autistic individuals tend to differentiate more, but generalize less strongly between stimuli, leading to a more detail-focused processing style. In light of the evidence, the original WCC theory was honed to “detail-focused cognitive style” (Happé and Frith, 2006).

In summary, the preference for a local processing mode (if not otherwise instructed) by autistic individuals stands in contrast to that by TD individuals, who tend to show a global processing preference (if not otherwise instructed). This raises the intriguing question of whether the respective default processing style might be influenced dynamically under certain task designs. For instance, it has been shown that participants with ASD can process globally if explicitly instructed to do so, e.g., in a selective attention task (Plaisted et al., 1999; Mottron et al., 2003).

Priming is a traditional technique to modify individual differences in perception despite identical stimulus material. It is used to analyze the influence of situational variables on perception, cognition, feelings and behavior (Bargh and Chartrand, 2000). Indeed, a whole series of studies has demonstrated the effect of priming on the visuo-spatial perception of TD participants (e.g., Lin et al., 2008). Many researchers are of the opinion that situational factors in daily life trigger similar priming effects and therefore have a significant influence on our perception (Kühnen et al., 2001; Kühnen and Oyserman, 2002; Lin et al., 2008; Oyserman and Lee, 2008; Oyserman et al., 2009). However, to date, research on ASD and priming is sparse. Thus, the aim of the current study was to test, whether a processing preference can be overcome through priming in ASD as in TD.

Some studies have already employed priming tasks in autistic people. Semantic priming makes use of a semantic relationship between the content being primed and the task that follows. It has been used in some studies to explore the spontaneous use of a contextual, i.e., global, processing mechanism in autistic and TD individuals (Kamio and Toichi, 2000; Toichi and Kamio, 2001; López and Leekam, 2003; Hala et al., 2007; Kamio et al., 2007). Toichi and Kamio (2001) examined the ability of autistic individuals to use semantic connections to complete words. The semantic priming was performed by presenting a semantically related word to the target word. Autistic and TD individuals showed comparable abilities in completing target words. As in the TD group, the autistic individuals performed worse if there was no semantic connection between the priming word and the target word. Thus, all participants, irrespective of diagnosis, showed no differences in receptivity to a semantic priming (Toichi and Kamio, 2001). Interestingly, Kamio and Toichi (2000) differentiated in their study between two different forms of semantic priming: they used either related words or images as references to the target word to be completed. While control persons showed a comparable improvement after both priming methods, autistic individuals were significantly better when image priming was used compared to word priming. Kamio et al. (2007) examined autistic individuals without speech development delay in a similar paradigm. Using semantically close words, a priming was performed, in which participants were asked to decide whether a target word is an existing word or not. While control persons performed better after priming, no priming effect was observed in the autistic participants. This contrasts with the previously mentioned studies of the same team, rendering the literature on semantic priming in autism inconclusive.

While semantic priming lends itself to language studies, the present study set out to investigate procedural priming, which assumes that the use of a cognitive procedure on a given task can heighten the likelihood that the same procedure being used on a subsequent task. To test the modifiability of a default processing style we made use of a figure-disembedding task, in which autistic individuals have previously been shown to have a superior performance to TD individuals (Falter et al., 2008). This task requires participants to detect a smaller shape as a constituent part of a more complex figure. For procedural priming preceding the figure-disembedding task, we used the Navon task (Navon, 1977) requiring either local or global processing, as already used for priming purposes before (Förster et al., 2008). We therefore investigated whether procedural priming of global processing (henceforth: global priming) might deteriorate performance on a figure-disembedding task in autistic individuals compared to their baseline performance, which has been found to show a local processing tendency in previous perceptual studies (e.g., Falter and Bailey, 2011; Falter, 2012; Falter et al., 2013). We also investigated whether procedural priming of local processing (henceforth: local priming) might improve performance in TD individuals compared to their baseline performance. The potential of dynamically changing processing styles on figure-disembedding would show the extent to which the alleged processing differences in ASD compared to TD are modifiable by context and task design.

First, in concordance with findings by Muth et al. (2014), we hypothesized (H1) that the performance on the figure-disembedding task would be superior in autistic compared to TD individuals in the unprimed condition (i.e., baseline). Second, given the idea of a general default tendency toward a local processing style in ASD and global processing style in TD, we predicted (H2) reduced performance after global priming in the autistic group and (H3) increased performance after local priming in the TD group, whereas priming modes that already fit with the general tendency should not make a difference in performance in either group.

## Materials and Methods

### Participants

A total of 29 autistic participants and 22 TD adult control participants took part in the study. Technical issues resulted in incomplete data sets of four ASD and one TD participant. The analyzed samples therefore included 25 evaluable ASD data sets (16 males, 9 females) and 21 TD data sets (8 males, 13 females). All participants were between 24 and 56 years old. The autistic individuals were older (M = 47.48, SD = 6.56) than TD individuals (M = 40.24, SD = 7.58). However, the groups were matched by verbal IQ, performance IQ, and full IQ (see Table 1 for descriptive statistics and matching).

**Table 1:** Demographic data.

	TD (*n* = 21)		ASD (*n* = 25^a^)		W	*p*-value	Rank biserial correlation
	Mean	SD	Mean	SD			
Age	40.24	7.77	47.48	6.70	409.00	0.001	0.56
BDI	2.52	2.75	13.00	8.13	479.50	<0.001	0.83
VIQ	110.86	17.00	116.76	17.11	320.50	0.204	0.22
PIQ	103.33	15.88	111.68	19.26	345.00	0.070	0.31
FIQ	108.24	16.8	115.68	18.51	331.00	0.133	0.26
AQ	14.62	4.59	43.16	2.76	525.00	<0.001	1.00
EQ	50.67	13.79	14.38	7.62	5.50	<0.001	−0.98
SQ	22.24	8.16	43.25	14.01	441.00	<0.001	0.75

Mann Whitney *U*-test was performed, because most variables violated the assumption of normality (determined by Shapiro–Wilk test). Rank biserial correlation is a method of *reporting* the effect size for the Mann*–*Whitney *U-*test. BDI, Beck’s Depression Inventory; VIQ, verbal intelligence quotient; PIQ, performance intelligence quotient; FIQ, full intelligence quotient; VIQ, PIQ, and FIQ were measured using the Wechsler Adult Intelligence Scale. AQ, Autism Quotient; EQ, Empathy Questionnaire; SQ, Systemizing Questionnaire. Standard deviations (SD), scores for both the Autism Spectrum Disorder (ASD) group and the typically developed (TD) group.

a *n = 24* for EQ and SQ.

Participants were recruited through the Autism Outpatient Unit of the University Hospital Cologne. Study inclusion criteria for the TD group were lack of a neurological or psychiatric diagnosis, no use of psychotropic medication, normal or corrected-to-normal vision and age between 18 and 60 years. Study inclusion criteria for the ASD group were a diagnosis of autistic disorder (F84.0) and Asperger’s Syndrome (F84.5) according to ICD-10 [World Health Organization (WHO), 1993], with an at least average Full Scale IQ (FIQ > 85). Further, participants were included if they had no comorbid neurological or psychiatric diagnosis, no use of psychotropic medication, and if they had normal or corrected-to-normal vision and age between 18 and 60 years. Participants took part in an unrelated study on alexithymia and emotion recognition on the same testing day (unpublished data). The study was approved by the ethics committee of the Medical Faculty of the University of Cologne. Participants provided consent prior to initiating the study.

### Neuropsychological Tests and Questionnaires

#### Wechsler Adult Intelligence Scale—Fourth Edition

The cognitive abilities were measured with the Wechsler Adult Intelligence Scale*—*Fourth Edition (WAIS-IV, Petermann, 2012). There were no significant differences between the groups for either performance or verbal IQ (see Table 1).

#### Beck Depression Inventory—II

The *Beck Depression Inventory—II* (BDI-II, Beck et al., 1996), was used to measure the severity of depressive symptomology. The reasoning for this is because depression is a common comorbidity in autism (e.g., Hollocks et al., 2019) and it is associated with differences in motivation and cognition (Grahek et al., 2019). The participants with ASD had a significantly higher BDI score (see Table 1).

#### Autism Spectrum Quotient Test, Systemizing Quotient, and Empathy Quotient

The *Autism Spectrum Quotient Test* (AQ, Baron-Cohen et al., 2001), *The Systemizing Quotient* (SQ, Baron-Cohen et al., 2003) and *The Empathy Quotient* (EQ, Baron-Cohen and Wheelwright, 2004), were used to measure traits associated with ASD. One participant with ASD had not filled out the EQ and the SQ. As expected, the AQ and SQ scores of the ASD participants were significantly higher and the EQ score of the ASD participants significantly lower than the control scores (see Table 1). All control participants achieved an AQ score below the CUT-OFF value of ≥32 points. For demographic data see Table 1.

### Design, Task, and Procedure

The study has a mixed 2 × 3 design with a between-subject factor Group (with two levels: ASD or TD) and a within-subject factor Priming (with three levels: baseline, local or global). The Navon Figures (NF) task was used for priming (Navon, 1977). The dependent variable was the performance (accuracy and reaction times) on a computerized version of a figure-disembedding task, known as the Embedded Figures Task (EFT) to measure the priming effect on visual perception (Falter et al., 2006, 2008). Each of these two tasks (Navon Figures for priming and EFT for measuring visual perception) are detailed below, after which the general procedure is outlined.

**Figure 1:**
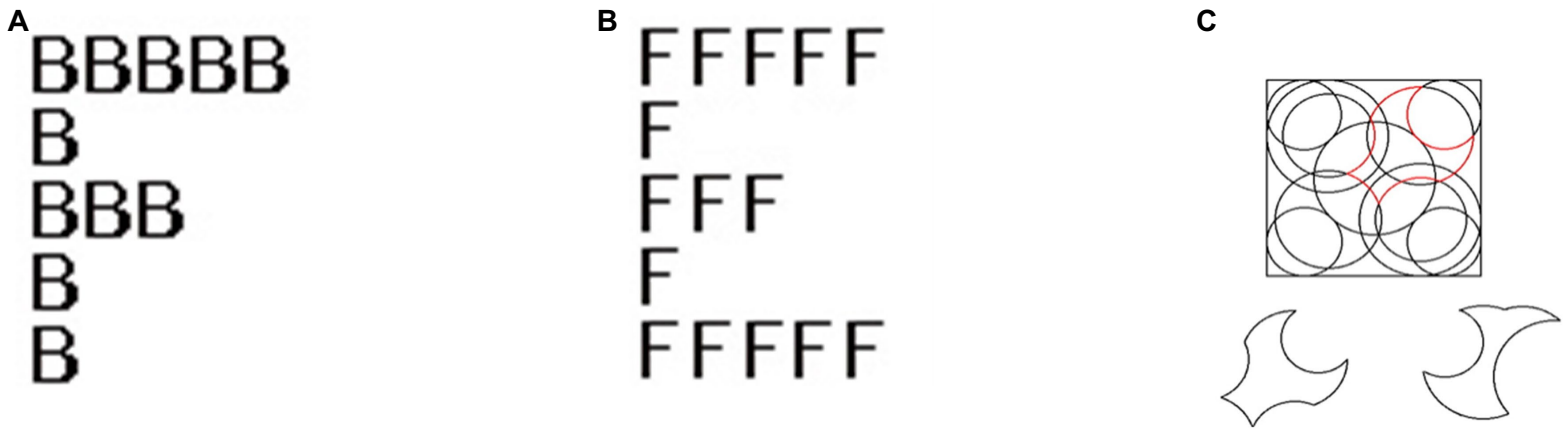
Sample stimuli or Navon Figures (NF) for priming. Instruction: “Does the stimulus contain an F or an H?”; **(A)** global priming, **(B)** local priming, and **(C)** presents an example stimulus of the Figure-Disembedding Task with a complex figure at the top (with shape inserted in red for illustration purposes) and two answer options at the bottom (the left shape would be the correct answer option).

The priming consisted of the Navon Figures task (Navon, 1977). NFs consisted of small letters (0.5°–0.5° of visual angle), which were grouped to form large letters (2.5°–2.5° of visual angle). The idea behind them, namely that small letters are arranged to form large letters, offer both local and global features to be processed. The NFs always contained the target letters “F” or “H” (see Figure 1). In the “local” condition, one of the two target letters corresponded to the small letters, and in the “global” condition one of the two target letters corresponded to the large letters. There were 14 different stimuli for the local and 14 for the global priming condition. These stimuli were shown repeatedly, in randomized order, so that each participant worked on a total of 72 NFs per condition. Participants were instructed to identify as quickly but as accurately as possible, which of the two target letters was presented and to press the “F” or “H” keys accordingly. The progression was self-paced. The number of correct answers and the corresponding reaction times were recorded. It is important to highlight at this stage that the “baseline” condition was one where there was no priming and hence no Navon task to complete at all. It was always the first block to complete.

A computerized version of a figure-disembedding task (i.e., EFT), was used to measure visual perception before and after priming, which has already been successfully used in several previous studies (Falter et al., 2006, 2008; Brosnan et al., 2012). Each trial consisted of a colored, complex image and two shapes below it. The participants were asked to decide as quickly but as accurately as possible which of the two shapes was embedded in the complex image. The progression was therefore self-paced (see Figure 1B). They were instructed to give their answers by pressing one of two keys: the “F” key for the left form, the “H” key for the right form. In half of the trials the right shape was embedded, in the other half the left shape. Auditory and visual feedback was given for 2,000 ms; two different cues for a correct and a wrong answer were played (auditory) and the embedded shape was marked in the complex picture by a border (visual).

Regarding the overall procedure, after completing the questionnaires and going through the instruction for the main task, the presentation of the above-mentioned tasks was as follows. Participants worked through two practice trials requiring figure-disembedding. The inter-trial interval was always 500 ms. The first block was always unprimed and consisted of six figure-disembedding trials to measure a baseline performance against which to compare each of the two priming blocks to follow. The second and third blocks were primed with a priming condition using the NF task (local then global or *vice-versa*, counterbalanced across participants within each group). The priming condition (local or global) remained the same within a block. The second block started with 48 Navon priming trials (either local or global, depending on the condition) followed by six figure-disembedding trials. Before each figure-disembedding trial, an additional four priming trials were presented in order to prevent the priming effect from fading. The third block had the same structure as the second block, except that the alternative priming condition (local or global, respectively) was presented preceding the figure-disembedding trials. The allocation of figure-disembedding trials to the local, global or unprimed condition was randomized for each individual experimental run.

The total duration of the study, including the questionnaires was about 60 min. At the end participants were debriefed and paid for their participation. To keep the distance between eye and screen constant at 59 cm a chin and forehead support was used. The stimulus presentation and response recording in both tasks were performed by the software package Presentation (version 12.2; Neurobehavioral Systems, Inc.).^1^

### Statistical Analysis

As data was collected as repeated measures within subjects, and considering that the variance in individuals and items is clustered, mixed effects models have been fitted in R (R Core Team, 2019). These are a robust alternative to Analyses of Variance (Baayen et al., 2008; Jaeger, 2008; Singmann and Kellen, 2019). To account for the nested data structure, random intercepts have been modeled for variance clustered in subjects and stimuli. For accuracy (i.e., binary response variable) generalized linear mixed effects models have been fitted using the *lme4* package in R (Bates et al., 2014). Model diagnostics have been visually inspected for all models. Likelihood ratio tests were calculated to test if a predictor in question significantly explained variance in the response variable against an alpha level of 0.05. Important R packages that were used during mixed models analysis were *afex* (Singmann et al., 2015) and *performance* (Lüdecke et al., 2021).

## Results

Descriptive statistics can be found in Table 2.

**Table 2:** Reaction time (ms) and accuracy by participant group.

	TD			ASD				
	Mean	SD	Min	Max	Mean	SD	Min	Max
ACC baseline	3.43	1.40	1.00	6.00	4.16	0.75	3.00	5.00
ACC global	4.29	1.35	2.00	6.00	4.16	1.28	2.00	6.00
ACC local	4.00	1.18	1.00	6.00	4.04	1.27	2.00	6.00
RT baseline	12042.86	11474.65	2447.00	51050.00	14473.08	12221.67	2167.00	58569.00
RT global	10358.52	7162.06	2363.00	35832.00	12593.44	9343.76	2705.00	41895.00
RT local	12376.76	8436.30	3079.00	37127.00	13056.12	10100.69	2866.00	45205.00

Reaction times (RT) in milliseconds and accuracy scores (ACC) for baseline, globally primed (global) and locally primed (local) conditions. Standard deviations (SD), minimum (Min) and maximum (Max) scores for both the Autism Spectrum Disorder (ASD) group and the typically developed (TD) group.

### Baseline Condition

To test H1, investigating a group difference in the baseline condition, we found significantly superior accuracy of participants with ASD compared to TD individuals using directed Mann Whitney U-test due to singular model fit: [*W*(44) = 348, *p* = 0.025, *r* = 0.290].There was no group difference concerning RT (we performed the analysis only for the RTs in correct trials) in the baseline condition [W(44) = 308, *p* = 0.162] Thus, we concentrated on accuracy data in the following analyses and are reporting the non-significant results of the analyses for the RTs in the Supplementary Materials.

### Priming Effect

Testing for H2, a global priming effect, first, we fitted models for data from baseline and global conditions only. We included fixed effects for Group (ASD; TD), Condition (baseline; global), and their interaction. We found a trend of a main effect of Condition [*χ*^2^(1) = 3.19, *p* = 0.074], but in the non-expected direction, with greater accuracies in the global condition compared to baseline. There was no significant main effect for Group [*χ*^2^(1) = 1.05, *p* = 0.305], but a trend of an interaction [*χ*^2^(1) = 3.22, *p* = 0.073], indicating that global priming rather improved accuracies in the TD group.

Testing for H3, a local priming effect, we fitted models for data from baseline and local conditions only. We included fixed effects for Group (ASD; TD), Condition (baseline; local), and their interaction. Results showed no main effects of Condition [*χ*^2^(1) = 1.05, *p* = 0.305], Group [*χ*^2^(1) = 2.05, *p* = 0.152], and their interaction [*χ*^2^(1) = 1.61, *p* = 0.204].

Given a trend to better performance in the global priming condition compared to the baseline condition, despite of the expected detrimental effect of priming toward a global processing style, we had to assume a training effect, which was analyzed in the following.

### Training Effect

**Figure 2:**
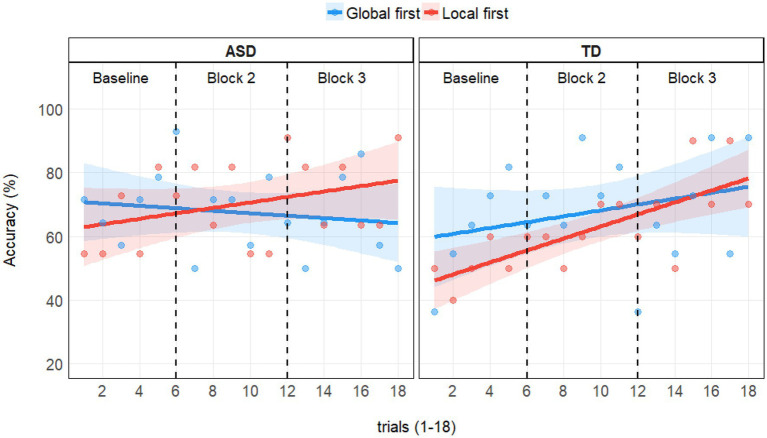
Average accuracies per group (ASD left panel; TD right panel) for each trial (1–18) depending on priming condition order in the Embedded Figure Task (EFT). One point depicts the percentage of accurate responses per trial number in groups (ASD left panel; TD right panel) and condition orders (global first in blue; local first in red). Linear regression lines with confidence bands fitted per group and condition order combination. Black dashed lines illustrate the block limits: Trials 1–6 were baseline trials, which were followed by two blocks each with 6 trials that included global or local priming in a counterbalanced order.

**Figure 3:**
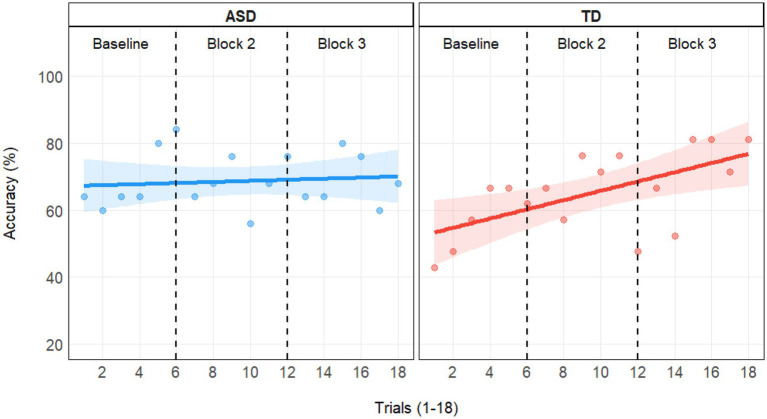
Average accuracies per group (ASD left panel; TD right panel) for each trial (1–18) in the Embedded Figure Task (EFT). One point depicts the percentage of accurate responses in the Embedded Figures Task per trial number in groups (ASD left panel; TD right panel). Linear regression lines with confidence bands fitted per group. Black dashed lines illustrate the block limits: Trials 1–6 were baseline trials, which were followed by two blocks each with 6 trials that included global or local priming in a counterbalanced order.

We ran a model with accuracy predicted by Trialnumber, Group, and their interaction and found a significant effect of Trialnumber [*χ*^2^(1) = 6.96, *p* = 0.008], indicating improvement in performance over time. Group was non-significant [*χ*^2^(1) = 0.63, *p* = 0.427], but the interaction showed that there was a trend indicating a larger training effect for the TD group [*χ*^2^(1) = 3.51, *p* = 0.061]. Figure 2 depicts accuracies in percentage per trial numbers in groups as well as priming condition orders. The positive slopes show an increase in accuracies over time, which was especially pronounced in the TD group. The training effect pooled for both priming condition orders is best visualized in Figure 3.

### Covariates

**Figure 4:**
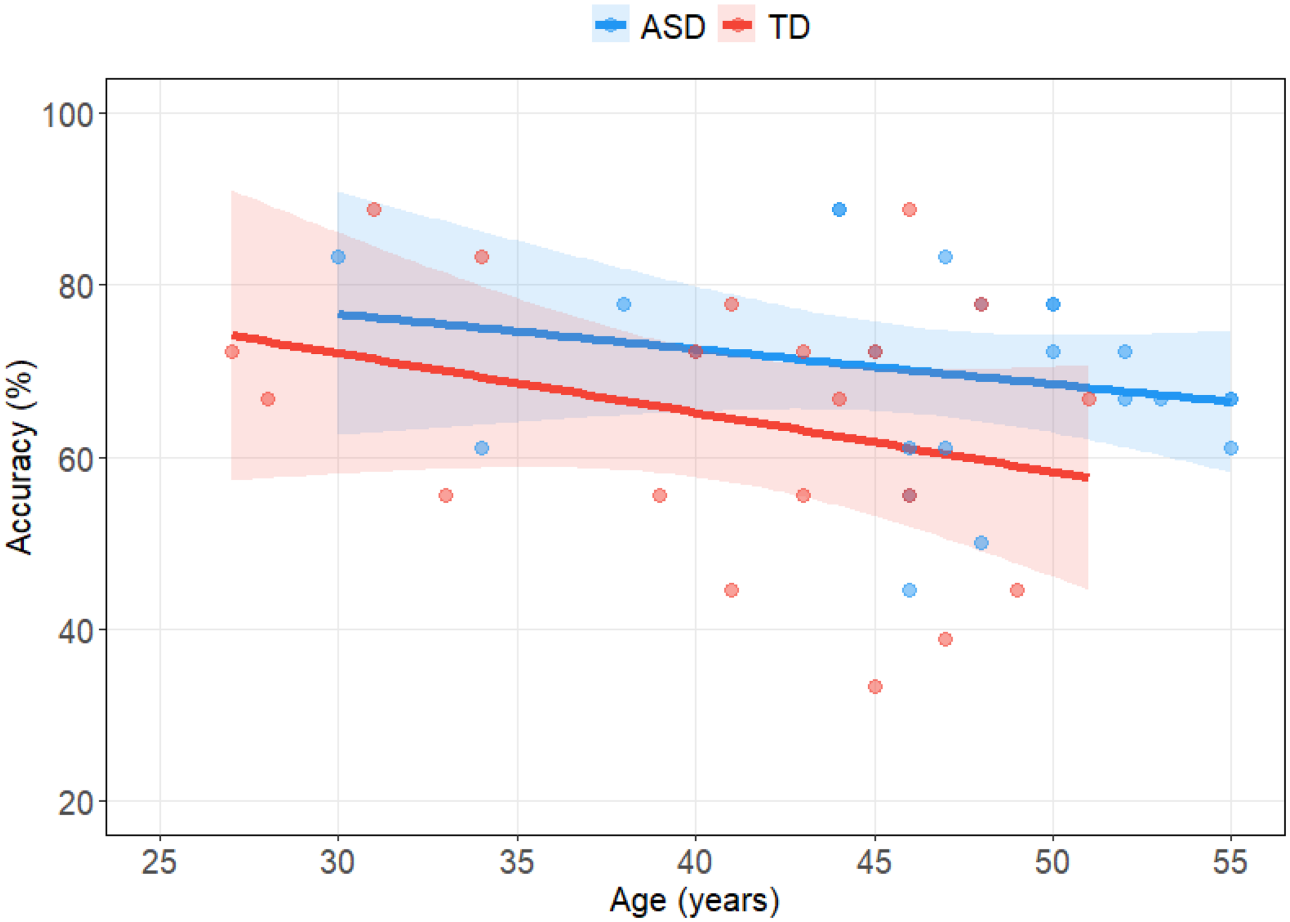
Correlations of age and average accuracies in Embedded Figure Tasks (EFT) in ASD group (blue) and TD group (red). One point represents the age and mean accuracy over all Embedded Figure Task (EFT) trials of one participant. Linear regression lines with confidence bands depict negative slopes in both groups.

We tested for potential effects of FIQ, AQ, BDI, and age on performance. We found a significant effect of FIQ [*χ*^2^(1) = 15.96, *p* < 0.001], and a significant interaction effect between FIQ and Group [*χ*^2^(1) = 4.52, *p* = 0.033] showing a stronger increase in EFT accuracies with increasing FIQ in the TD group. In order to analyze whether the training effect might be affected by the influence of FIQ we have run a full model including FIQ, Trialnumber, as well as Group as fixed factors with their interaction terms and found that the two-way interaction of FIQ and Trialnumber was non-significant [*χ*^2^(1) = 0.30, *p* = 0.584] as well as the three-way interaction with Group * FIQ * Trialnumber [*χ*^2^(1) = 0.31, *p* = 0.579]. The main effects for Trialnumber [*χ*^2^(1) = 7.51, *p* = 0.006] and FIQ [*χ*^2^(1) = 16.23, *p* < 0.001] remained significant, as well as the interaction terms Trialnumber * Group [*χ*^2^(1) = 4.01, *p* = 0.045] and FIQ * Group [*χ*^2^(1) = 4.82, *p* = 0.028]. Additionally, in a model containing Age, Group, and their interaction, we found a main effect of Age [*χ*^2^(1) = 5.60, *p* = 0.018] and a trend of Group [*χ*^2^(1) = 3.66, *p* = 0.056]. A non-significant interaction term showed that the effect of age was similar across both groups [*χ*^2^(1) = 0.04, *p* = 0.840]. The effect of age did not cause the superior performance of the ASD group in the baseline condition, but if anything would have made the effect more conservative (see Figure 4). In a model containing BDI, Group, and their interaction, we found that BDI did not explain variance in accuracies above chance level [*χ*^2^(1) = 0.07, *p* = 0.795], neither did group [*χ*^2^(1) = 0.06, *p* = 0.812], and the interaction of BDI and Group [*χ*^2^(1) = 0.11, *p* = 0.735].

## Discussion

The support for H1, namely superior accuracy in participants with ASD in the unprimed baseline condition corroborates previous findings (e.g., Shah and Frith, 1983; De Jonge et al., 2006; Pellicano et al., 2006; Brosnan et al., 2012; Muth et al., 2014). However, other studies also show equal performance (e.g., Rodgers, 2000; Bölte et al., 2007; Kaland et al., 2007; Chen et al., 2008; White and Saldaña, 2011) as summarized in a recent systematic review (Horlin et al., 2016). Considering this discrepancy in findings, the results of the current study that control participants’ performance can be improved by training is highly relevant and any findings of superior or equal performance in figure-disembedding needs to be evaluated on the basis of potential differential impact of training on group averages.

Against our expectations (H2), we did not find a deterioration of performance after global or local priming in both groups (H3). A performance improvement trend in the TD group irrespective of priming points to a training effect, which was indeed confirmed in a multilevel model analysis. This was not visible in the RT data, possibly due to the fact that our instruction to participants was “as quickly but as accurately as possible” is highlighting the importance of accuracy.” Notably, neither a performance improvement nor decrease was observable in the ASD group, which renders the ASD group immune against either priming or training effects in this task. It is possible that either ASD performance is very stable and robust or that ASD individuals perform at ceiling und we therefore cannot observe a training effect. In the case of the first possibility, this would be in concert with the literature showing that ASD individuals tend to take context less into account (i.e., WCC or EPF theories mentioned in the introduction).

Our results are in accordance with previous studies that did not find any priming effect in ASD employing semantic priming (Kamio et al., 2007) but contrast others that did show semantic priming effects (Kamio and Toichi, 2000). In addition, the type of priming used in the current study, has been used in previous research (Förster et al., 2008) and was effectively a sequential priming task, for which it has been shown that target-level information (i.e., global or local priming in hierarchical stimuli) mediate priming effects in subsequent trials (Kim et al., 1999). Thus, not just the priming type but also the priming procedure used in the current study was markedly different from the semantic priming tasks described above. Yet, our results are also in line with previous experiments showing a lack of configural priming in individuals with ASD (Behrmann et al., 2006) with a sequential and non-semantic approach.

We found a significant influence of IQ on performance and improvement by training in the TD group only. Arguably, the higher the cognitive abilities the faster improvements by learning occur. Interestingly though performance in the ASD group was stable throughout. A further significant influence was found by age with decreasing performance the older the participants were. The effect of age could not have influence our findings of superior baseline figure-disembedding in ASD, because our ASD group was somewhat older than the TD control group. Thus, our results could if anything be regarded as conservative and potentially decreasing the group difference.

The findings of this study have to be interpreted in light of some limitations. First, the number of trials per condition in the figure-disembedding task was limited. However, increasing the trial number would potentially cause fatigue in participants. Future research should attempt to replicate the findings with larger trial numbers and possibly a between-subject design regarding the priming factor. Second, we provided feedback on the disembedding task, which might have contributed to the training effect. However, the feedback was unique to each trial and not strategy based, therefore we do not believe that knowing whether or not one was correct or incorrect, would have affected their performance. At most, it could have (de)motivated participants making errors over time. Third, we did not perform a sample size estimation, due to the fact that there were no previous effects to base this on and it is possible that we had too little power to detect priming effects in this study. Future research should attempt to replicate the procedure with larger samples. Further, the study sample were adults with ASD without intellectual disabilities limiting generalizability within the spectrum given that up to 50% of autistic disorders have an intellectual disability (Charman et al., 2011). Finally, the use of NF for procedural priming can be implemented in different ways. In the current study, we used a previously used approach (Förster et al., 2008). In other versions of priming with NF participants had to search at the local or global level to respond. For example, Tan and colleagues (Tan et al., 2017) did not use target letters, but merely instructed participants to attend to either the large or the small letters (see also Macrae and Lewis, 2002; Förster and Higgins, 2005). In yet another version (Gao et al., 2011), two Navon stimuli were presented to participants simultaneously in the left and right visual field, respectively, and participants were instructed to match the two displays, focusing either on the large letters, ignoring the small letters (global level), or on the small letters, ignoring the large letters (local level). Future research should attempt to replicate our findings using other procedural priming tasks, in particular Navon variations.

In conclusion, figure-disembedding seems to be a function of visuo-spatial cognition that shows solid high performance in individuals with ASD without modifiability through priming or training. In contrast, performance in control participants was improved to the level observed in ASD by training. These results imply that any findings of superior performance in figure-disembedding tasks in ASD should take the potentially differential influence of training into account and carefully examine whether the initially found superiority effect holds throughout the experimental trials performed or might lead to equal performance between groups.

## Data Availability Statement

The raw data supporting the conclusions of this article will be made available by the authors, without undue reservation.

## Ethics Statement

The studies involving human participants were reviewed and approved by Ethics Committee of the Medical Faculty of the University of Cologne. The patients/participants provided their written informed consent to participate in this study.

## Author Contributions

CF and AG conceptualization; LH data curation; CB, CF, AG, LH and MR formal analysis; KV funding acquisition; CF, AG, LH investigation; CF and AG methodology; CF, AG, and KV project administration; KV resources; CF, AG, and KV supervision; CF, LH, and MR original draft; CF, AG, MR, CB and KV review & editing. All authors contributed to the article and approved the submitted version.

## Funding

CB, MR, and CF were supported by the DFG (German Research Council; grant numbers: FA 876/3–1 and 876/5–1). KV was supported by the DFG (CRC 1252) and by the EC, Horizon 2020 Framework Programme, FET Proactive (Project VIRTUALTIMES; Grant agreement ID: 824128).

## Conflict of Interest

The authors declare that the research was conducted in the absence of any commercial or financial relationships that could be construed as a potential conflict of interest.

## Publisher’s Note

All claims expressed in this article are solely those of the authors and do not necessarily represent those of their affiliated organizations, or those of the publisher, the editors and the reviewers. Any product that may be evaluated in this article, or claim that may be made by its manufacturer, is not guaranteed or endorsed by the publisher.

## Footnotes

^1^ www.neurobs.com/
