# Supplementary material for: Figure-Disembedding Is Inferior in Non-autistic Compared to Autistic Individuals but Can Be Improved by Training
Source: Front Psychol. 2022 Jul 25;13:857630. doi: 10.3389/fpsyg.2022.857630 (PMC9358439; doi:10.3389/fpsyg.2022.857630)
Supplement: Supplementary file 1 [file Data_Sheet_1.docx]

RT Analysis

**Only correct trials (n = 555 correct trials in total): ASD n = 309; TD n = 246**

**Figure 1.** Density plots in groups with RTs of correct trials.


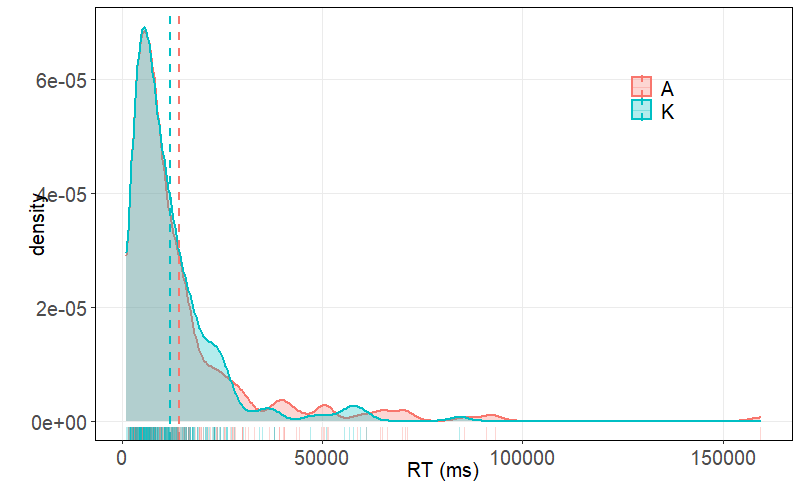


*Note*. Outliers result in skewed data. 13 values with RT > 1 minute will be removed in a next step.

**Figure 2.** Density plots in groups with RTs of correct trials after removal of 13 outliers (RT > 1 minute).


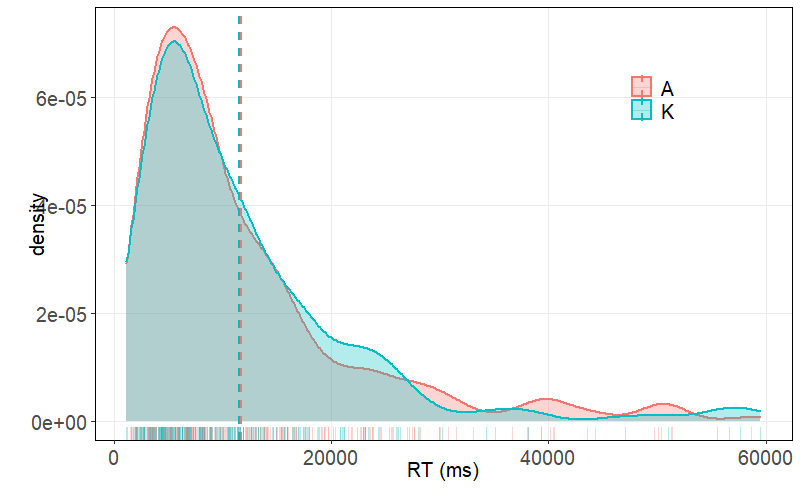


*Note*. Trimmed data looks more decent but is still skewed, as it is typical for RT data. In a next step log-transformation will be applied.

**Figure 3.** Density plots in groups with RTs of correct trials after removal of 13 outliers (RT > 1 minute) and log-transformation of the RTs.


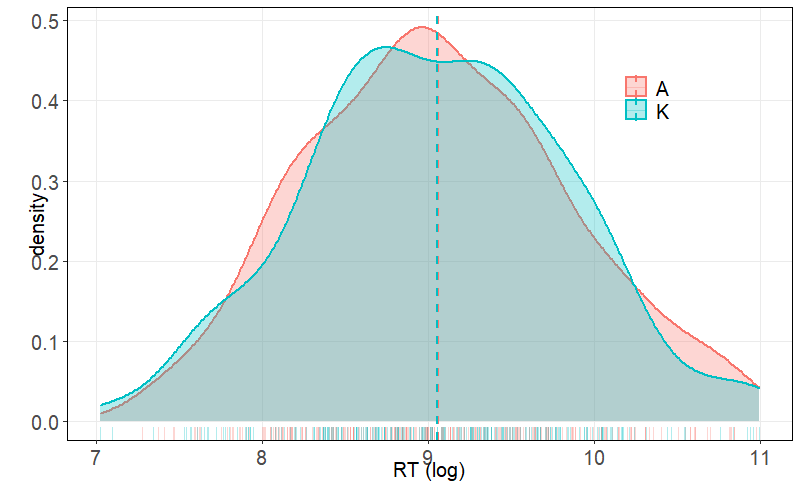


*Note*. All linear mixed models will use the log-transformed RT as response variable.

**ANALYSIS 1)**

QUESTION: *Is there a group difference in contrast global:baseline?*

MODEL: RT_log ~ GROUP * CONDITION + (1 | ID) + (1 | STIM), data = data.NG (Please note that the abbreviation L stands for local and the abbreviation N for native i.e. baseline)

OUTPUT:

Effect df Chisq p.value

1 GROUP 1 0.04 .834

**2 CONDITION 1 3.14 + .076**

3 GROUP:CONDITION 1 0.02 .894

RESULT: Marginal effect for condition, with rather slower responses in baseline:


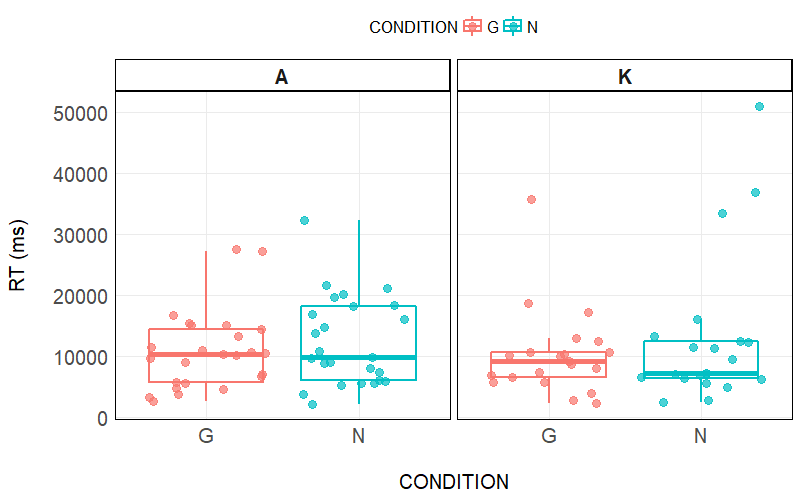


**ANALYSIS 2)**

QUESTION: *Is there a group difference in contrast local:baseline?*

MODEL: RT_log ~ GROUP * CONDITION + (1 | ID) + (1 | STIM), data = data.NL (Please note that the abbreviation L stands for local and the abbreviation N for native i.e. baseline)

OUTPUT:

Effect df Chisq p.value

1 GROUP 1 0.00 .951

2 CONDITION 1 0.26 .612

3 GROUP:CONDITION 1 0.19 .660

RESULT: No effects in comparison of baseline and local priming trials.

**ANALYSIS 3)**

QUESTION: *Is there an equal training effect in groups?*

MODEL: RT_log ~ GROUP * trialnumber_centered + (1 | ID) + (1 | STIM), data = df_RT_trimmed

OUTPUT:

Effect df Chisq p.value

1 GROUP 1 0.02 .893

**2 trialnumber_centered 1 2.76 + .097**

3 GROUP:trialnumber_centered 1 0.02 .890

RESULT: Marginal effect of trialnumber, with increasing RTs in both groups.


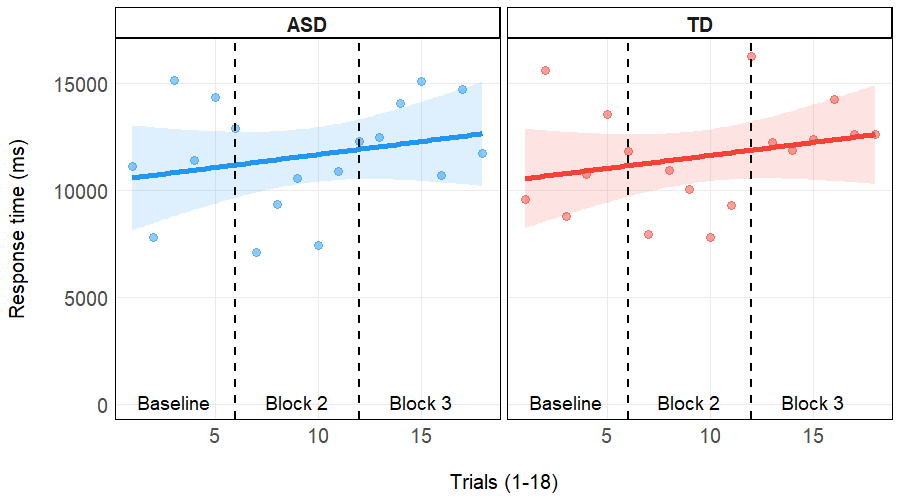


**ANALYSIS 4)**

QUESTION: *Does FIQ has an impact on performance in groups?*

MODEL: RT_log ~ GROUP * FIQ_centered + (1 | ID) + (1 | STIM), data = df_RT_trimmed

OUTPUT:

Effect df Chisq p.value

1 GROUP 1 0.00 .956

2 FIQ_centered 1 0.07 .785

3 GROUP:FIQ_centered 1 0.70 .401

RESULT: No effect of FIQ on RTs in groups.

**ANALYSIS 4)**

QUESTION: *Does Age has an impact on performance in groups?*

MODEL: RT_log ~ GROUP * Age_centered + (1 | ID) + (1 | STIM), data = df_RT_trimmed

OUTPUT:

Effect df Chisq p.value

1 GROUP 1 0.06 .802

2 Age_centered 1 0.12 .727

3 GROUP:Age_centered 1 0.18 .669

RESULT: No effect of age on RTs in groups.

**ANALYSIS 5)**

QUESTION: *Does BDI has an impact on performance in groups?*

MODEL: RT_log ~ GROUP * BDI_centered + (1 | ID) + (1 | STIM), data = df_RT_trimmed

OUTPUT:

Effect df Chisq p.value

1 GROUP 1 0.00 .998

2 BDI_centered 1 0.00 .981

3 GROUP:Age_centered 1 0.35 .556

RESULT: No effect of BDI on RTs in groups.

**ANALYSIS 6)**

QUESTION: *Does AQ has an impact on performance in groups?*

MODEL: RT_log ~ GROUP * AQ_centered + (1 | ID) + (1 | STIM), data = df_RT_trimmed

OUTPUT:

Effect df Chisq p.value

1 GROUP 1 0.57 .452

2 AQ_centered 1 0.65 .422

3 GROUP:Age_centered 1 0.12 .733

RESULT: No effect of AQ on RTs in groups.

**ADDITIONAL ANALYSIS 7) Speed/Accuracy Trade-Off**

QUESTION: *Do slower responses predict higher accuracies?*

MODEL: ACC ~ RT * GROUP + (1 | ID) + (1 | STIM), data = full_data

OUTPUT:

Effect df Chisq p.value

1 GROUP 1 0.86 .355

2 RT_centered 1 0.84 .359

3 GROUP:RT_centered 1 0.55 .456

RESULT: No association of RTs with accuracies.
